# Ebola Virus Imported from Guinea to Senegal, 2014

**DOI:** 10.3201/eid2306.161092

**Published:** 2017-06

**Authors:** Daye Ka, Gamou Fall, Viviane Cissé Diallo, Ousmane Faye, Louise Deguenonvo Fortes, Oumar Faye, Elhadji Ibrahim Bah, Kadia Mbaye Diallo, Fanny Balique, Cheikh Tidiane Ndour, Moussa Seydi, Amadou Alpha Sall

**Affiliations:** Centre Hospitalier Universitaire de Fann, Dakar, Senegal (D. Ka, V. Cissé Diallo, L. Deguenonvo Fortes, K.M. Diallo, C.T. Ndour, M. Seydi);; Institut Pasteur de Dakar, Dakar (G. Fall, Ousmane Faye, Oumar Faye, F. Balique, A.A. Sall);; Hôpital National Donka, Conakry, Guinea (E.I. Bah)

**Keywords:** Ebola, Ebola virus, imported, Senegal, Guinea, Ebola virus disease, viruses

## Abstract

In March 2014, the World Health Organization declared an outbreak of Ebola virus disease in Guinea. In August 2014, a case caused by virus imported from Guinea occurred in Senegal, most likely resulting from nonsecure funerals and travel. Preparedness and surveillance in Senegal probably prevented secondary cases.

Ebola virus disease (EVD) is a hemorrhagic fever caused by Ebola virus (EBOV); the mortality rate is high (*1*,*2*). EBOV was discovered in 1976, simultaneously in Zaire (now the Democratic Republic of the Congo) and Sudan (*3*,*4*). Since then, small to large outbreaks have occurred sporadically in the Democratic Republic of the Congo, Sudan, Gabon, Uganda, Côte d’Ivoire, and Congo (*5*–*7*). 

In March 2014, the World Health Organization (WHO) reported an EVD outbreak caused by Zaire EBOV in Guinea (*8*,*9*). The main feature of this outbreak was its extension into urban areas and neighboring countries (Liberia, Sierra Leone, Nigeria, Senegal, Mali). Ten countries on 3 continents were affected; 28,646 confirmed, probable, and suspected cases and 11,323 deaths were recorded. 

In August 2014, Senegal was the fifth country in Africa to be affected by imported EBOV (*10*). We described this case, the patient’s itinerary and epidemiologic links with confirmed case-patients in Guinea, and the evolution of the disease and the virus.

The patient was a 21-year-old man from Forecariah, Guinea, who had traveled by land to Senegal during the night of August 13–14, 2014. The date of his illness onset was August 16, 2014; symptoms were fever, vomiting, diarrhea, yellow or black feces, anorexia, and asthenia. On August 18, he visited a suburban medical center in the suburbs of Dakar, where he received treatment for malaria: quinine, antipyretic and antimicrobial medications, and intravenous rehydration. Diarrhea and vomiting stopped on day 4 after illness onset, but fever and asthenia persisted. On August 26, the patient was admitted to Fann Hospital, Dakar, with slight dehydration, fever (39.2°C), and herpetic lesions. Because no epidemiologic link with EVD was established, the patient was not isolated. 

On August 27, a total of 12 members of the patient’s family, all suspected of having EVD, were admitted to an Ebola treatment center in Conakry, Guinea; test results indicated EBOV positivity for 6. Epidemiologic investigation indicated that a member of this family had traveled to Dakar and was hospitalized. The Epidemic Management Committee set up by WHO in Guinea established an epidemiologic link between the patient in Fann Hospital and the confirmed case-patients in Guinea and quickly informed the health authorities in Senegal. The patient in Fann Hospital finally acknowledged that he had attended his uncle’s nonsecure funeral in Guinea on August 10, before coming to Dakar (Figure). 

**Figure:**
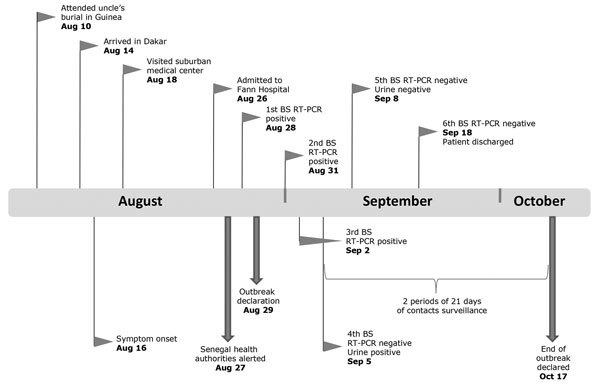
Timeline for case of Ebola virus disease imported into Senegal from Guinea, 2014. Flags indicate patient information; arrows indicate public health actions. BS, blood sample; RT-PCR, reverse transcription PCR.

On August 28, the man was transferred to an isolation center, and blood samples were sent to the Institut Pasteur laboratory in Dakar, a WHO-approved collaborating Centre for EBOV diagnostics. Real-time reverse transcription PCR (RT-PCR) was positive for Zaire EBOV; viral load was 2.04 × 10^4^ genome copies/mL. ELISA of the same sample detected Zaire EBOV–specific IgM (titer 1:400) and IgG (titer 1:3,200). This case of EVD in Senegal was reported to WHO on August 29. The patient received supportive care, and his clinical course progressed well; on August 31, he was apyrexic and his asthenia had decreased. In terms of virus evolution, a second blood sample tested on day 18 after illness onset showed diminution of viral load (4.96 × 10^3^ genome copies/mL) and an IgG titer increase to 1:6,400. A third blood sample collected on day 20 showed a negative RT-PCR result, but a urine sample collected the same day showed a positive result with a viral load of 2.04 × 10^4^ genome copies/mL. RT-PCRs of blood and urine collected on days 24 and 34 were negative, and serologic analyses showed a high IgG titer (1:12,800). 

The patient was declared cured on September 18, 2014. Epidemiologic investigations revealed a total of 74 contacts in Senegal, including 41 healthcare workers (from the suburban medical center and Fann Hospital). Symptoms developed in 5 of these contacts, but their test results were negative for EBOV. No secondary case was detected after 42 days of monitoring, and the outbreak in Senegal was declared over on October 17, 2014, with only 1 confirmed case reported.

The case-patient’s low viral load, detected during the first RT-PCR 10 days after illness onset, probably explains the absence of secondary cases in Fann Hospital. However, the absence of secondary cases in the suburban medical center that the patient had visited on days 3–4 after illness onset and among the family members in Dakar is a rare feature of EVD. The preparedness and surveillance established in Senegal after announcement of EVD in Guinea led to training of healthcare workers for proper use of protective equipment and security procedures with any patient, which probably prevented virus spread in the suburban medical center. This case of EBOV importation from Guinea to Senegal confirms the problems encountered with Ebola outbreak management, including the roles of nonsecure funerals and travel in virus spread.
